# Synthesis and Pharmacological Properties of Novel Esters Based on Monoterpenoids and Glycine

**DOI:** 10.3390/ph10020047

**Published:** 2017-05-18

**Authors:** Mariia Nesterkina, Iryna Kravchenko

**Affiliations:** 1 Department of Pharmaceutical Chemistry, I.I. Mechnikov Odessa National University, Odessa 65026, Ukraine; 2 Department of Organic and Pharmaceutical Technology, Odessa National Polytechnic University, Odessa 65044, Ukraine; kravchenko.pharm@gmail.com

**Keywords:** TRP channels, terpene esters, glycine, analgesic effect, anti-inflammatory action

## Abstract

Esters based on mono- and bicyclic terpenoids with glycine have been synthesized via Steglich esterification and characterized by ^1^H-NMR, IR, and mass spectral studies. Their analgesic and anti-inflammatory activities were investigated after transdermal delivery on models of formalin, capsaicin, and AITC-induced pain, respectively. Glycine esters of menthol and borneol exhibited higher antinociceptive action, whereas eugenol derivative significantly suppressed the development of the inflammatory process. The mechanism of competitive binding between terpenoid esters and TRPA1/TRPV1 agonists was proposed explaining significant analgesic effect of synthesized derivatives. For an explanation of high anti-inflammatory activity, competitive inhibition between terpenoid esters and AITC for binding sites of the TRPA1 ion channel has been suggested.

## 1. Introduction

The identification and structure determination of novel pharmacological targets—transient receptor potential (TRP) ion channels—provides an opportunity for the rational design and synthesis of TRP modulators. Numerous studies have shown that representatives of various TRP channel subfamilies are involved in pain perception and inflammatory processes [1,2,3]. In this context, TRPA1 and TRPV1 channels are of paramount interest to pharmacologists and practicing surgeons since antagonists of aforementioned receptors are considered promising drugs undergoing clinical trials [4]. These receptors have been established as molecular targets for a variety of phytochemicals among which special attention is paid to terpenes and their derivatives [5]. In addition to affecting the peripheral nervous system through interaction with TRP channels, terpene derivatives were found to act within the CNS as positive allosteric modulators of GABA_A_ receptors [6,7].

Recently, we have demonstrated the approach to obtaining compounds with combined pharmacological activity based on terpenoids and GABA [8]. The present paper is a logical continuation of our current research aimed at the search and study of compounds that are able to simultaneously bind to different types of receptors. Here, we focus on pharmacological properties of esters based on terpenoids and an amino acid neurotransmitter—glycine. The action of glycine within CNS, its co-release with GABA, and the co-activation of glycine and GABA receptors have already been sufficiently studied and described [9]. To date, of particular interest is the involvement of the glycinergic system in mechanisms of pain signal transmission and inflammatory processes [10,11]. Thus, the foregoing facts substantiate the feasibility of compound synthesis containing both glycine and terpenoids residues. In the present study, we investigated a range of pharmacological properties such as the analgesic and anti-inflammatory action of glycine esters with different terpenes.

## 2. Results and Discussion

### 2.1. Chemistry

**Scheme 1:**
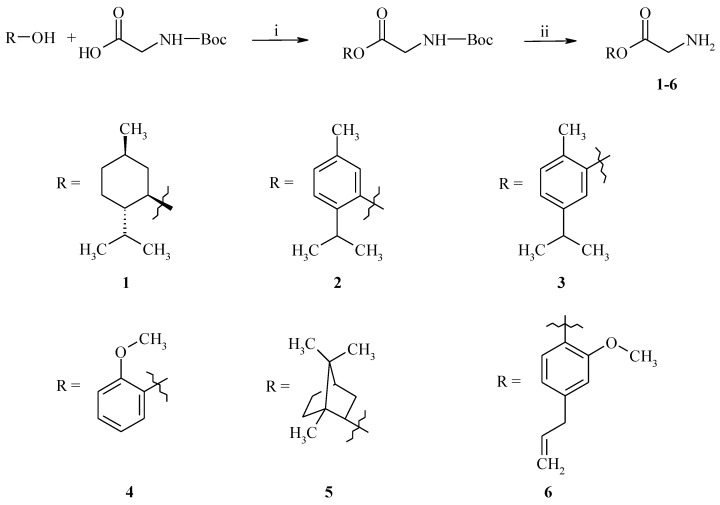
Synthetic pathway of Compounds **1**–**6**. *Reagents and conditions*: (i) DMAP, CH_2_Cl_2_, rt, 10 min; DCC, 0 °C, 30 min; rt, 10 h; (ii) HCl, CH_3_COOH. All esters were prepared as hydrochlorides.

Synthesis of esters based on mono- and bicyclic terpenes with glycine (**1**–**6**) was carried out via Steglich esterification with *N*,*N*′-dicyclohexylcarbodiimide (DCC) and 4-dimethylaminopyridine (DMAP) as a catalyst in dichloromethane (DCM). The following terpenes were used for ester preparation: L-menthol (**7**), thymol (**8**), carvacrol (**9**), guaiacol (**10**), borneol (**11**), and eugenol (**12**). Compound **1** has been prepared previously, but its pharmacological properties have not been sufficiently studied [12]. The condensation of 1.0 equivalent of an appropriate terpenoid with 1.05 equivalent of *tert*-butyloxycarbonyl (Boc)-protected glycine in the presence of DCC (1.1 equiv) and DMAP (catalytic amount) in dry DCM afforded the corresponding esters as yellow oily liquid (Scheme 1). The Boc group was removed with 1 M HCl in glacial acetic acid according to the literature procedure [12].

Synthesized esters of glycine were isolated as white or yellowish solids and fully characterized by ^1^H-NMR, IR-spectroscopy and FAB-mass spectrometry. The FAB-MS spectra of esters **1**–**6** display the protonated molecular ion peak [M + H]^+^ at *m*/*z* 214, 208, 208, 182, 212, and 222, respectively. Noteworthy, that mass spectra of Compounds **1** and **5** recorded by FAB-MS spectrometry contain additional peaks of dimeric and trimeric forms respectively at *m*/*z* 427 and 641 for ester **1** and at *m*/*z* 423 and 635 for ester **5**. The IR spectra of esters **1**–**6** exhibit absorption bands of N–H bonds (3326–3460 cm^−1^), C=O ester groups (1734–1783 cm^−1^), aromatic C–H (3020–3146 cm^−1^; 650–902 cm^−1^), and alkyl C–H (2851–2957 cm^−1^). The ^1^H-NMR spectral data contain resonance signals described by their chemical shift, integration, and multiplicity that are in full agreement with the presented molecular formulas. Thus, the structure of all synthesized esters is reliably confirmed by a series of spectral methods.

### 2.2. Pharmacology

#### 2.2.1. Antinociception Testing

There is strong evidence that TRPA1 and TRPV1 play a key role in nociception since the blockade of the abovementioned channels leads to reductions in pain sensation [4]. Moreover, TRPA1 and TRPV1 channels were found to simultaneously express in nociceptive nerves and physically interact with each other [13]. Terpenes and their derivatives comprise a large group of TRP modulators possessing analgesic action. Glycine receptors GlyT1, GlyT2, and GlyRs are also involved in inhibition of nociceptive signaling [10]. In order to elucidate the analgesic effect of synthesized esters based on terpenes and glycine, pharmacological models of thermal and chemical stimuli have been used. In our research, pain in experimental animals was caused by thermal stimuli in the “hot plate” test and by chemical stimuli via subplantar injection of formalin, capsaicin, and allyl isothiocyanate (AITC)—all of these pharmacological models are associated with the activation of TRP channels. Taking into account that TRP channels are expressed in various skin cells [14] and considering the ability of terpenes to fluidize lipids of the stratum corneum [15], esters **1**–**6** as well as the initial terpenoids—L-menthol (**7**), thymol (**8**), carvacrol (**9**) and guaiacol (**10**), borneol (**11**) and eugenol (**12**)—were delivered transdermally. The analgesic activity of compounds was compared with reference drug benzocaine (BZC), which was also found to activate TRPA1/TRPV1 channels [16].

In the “hot plate” test, mice that have been treated with the base ointment (control group) exhibited a mean reaction time (or latency response) of 10 ± 0.6 s (Table 1).

As illustrated in Table 1, when treated with the ointment containing either initial terpenoids or synthesized esters **1**–**6**, the reaction time significantly differed from the control group (*p* < 0.05 vs. control mice). BZC (positive control) was also found to increase the latency response to 18 ± 0.9 s; thus, esters **3** and **4** exert an antinociceptive effect similar to that in BZC treatment. When treated with Compounds **1**, **2**, **5**, and **6**, the reaction time ranged from 29 to 49 s, indicating that these compounds significantly attenuate thermally induced acute pain more than the reference drug BZC. Among all studied compounds, the highest threshold for painful thermal stimuli was observed for ester **5** as evidenced by the recorded latency response (49 ± 0.9 s).

Chemical stimulus wherein pain was caused by formalin, capsaicin, or AITC has been used as another pathway to induce acute pain in mice since all these pharmacological models are also associated with activation of TRP channels. Formalin-induced nociceptive response is well known to be biphasic reaction including direct activation of nociceptors (Phase I) and inflammatory/peripheral pain (Phase II) [17]. Until recently, the effect of Phase I was explained by C-fiber stimulation, but the specific molecular mechanism remained unclear. Detailed investigation of formalin-induced pain behavior demonstrated that formalin excites sensory neurons by action on TRPA1 ion channels via covalent modification of cysteine residues [18]. Aiming at a precise assessment of the response caused by the TRPA1 activation of C-fibers, we measured the licking time as an indicator of nociception only during Phase I.

Antinociceptive effect of Compounds **1**−**6** on Phase I of the formalin test in mice is represented in Table 2. A statistically significant decrease of paw licking time was observed in mice treated with esters **1**–**6**, initial terpenoids, and reference drug BZC (*p* < 0.01 vs. control mice). Licking time for Compounds **3**, **4**, and **6** ranges from 29 to 35 s, indicating that these compounds exert the same or a similar antinociceptive effect compared with BZC treatment (36 ± 2.4 s). The maximum analgesia after transdermal delivery was defined for alicyclic derivatives **1** (19 ± 3.7 s) and **5** (19 ± 1.5 s) containing residues of menthol and borneol, respectively.

Like formalin, another TRPA1 agonist allyl isothiocyanate is able to bind covalently to the channels leading to acute pain formation [13]. To justify the contribution of the TRPA1 receptor to the analgesic activity of synthesized esters **1**–**6**, AITC was used as a chemical stimulant (Table 3).

As seen, application of ointment base followed by the intraplantar injection of AITC elicited a nociceptive paw licking response in experimental animals with a duration of 71 ± 1.8 s, while for local anesthetic BZC this value was 48 ± 2.0 s. Compared with the data of the formalin test, a similar tendency is maintained for acyclic derivatives **1** and **5** as well as for initial terpenoids **7** and **11**—a statistically significant difference was observed between the aforementioned compounds and the reference drug BZC (*p* < 0.05). Furthermore, esters **2** and **3** differing by the substituent position in the aromatic ring were also found to better attenuate the pain reaction in comparison with BZC. Taken together, the ability to increase pain tolerance after transdermal delivery of glycine esters **1**–**6** has been confirmed using TRPA1 agonists—formalin and allyl isothiocyanate.

Despite the key role of TRPA1 receptors in pain perception and transmission, the strong interaction between TRPA1 and TRPV1 channels should be taken into account. As described above, AITC activates TRPA1 receptors, but mustard oil containing mainly AITC may also directly target TRPV1 receptors. Considering the cooperation between these receptors TRPV1 agonist capsaicin was also used in this study to induce acute pain (Table 4).

It is noteworthy that esters **1**–**6** suppress the pain reaction induced by subplantar capsaicin injection on average by three times in comparison with the control group of animals (46 ± 1.8 s). When comparing the licking time of mice treated with menthol **7** and ester **1**, a statistically significant difference was observed between these compounds and reference drug BZC (*p* < 0.05). Generally, we may conclude that esters based on terpenoids with glycine exhibit similar or higher antinociceptive action in comparison with BZC after transdermal delivery as evidenced by lower licking time recorded for these compounds. Thus, competitive binding between terpenoid esters and TRPA1/TRPV1 agonists might be proposed explaining a significant analgesic effect of synthesized derivatives. Moreover, these findings serve as a corroboration of the interaction between TRPA1 and TRPV1 ion channels.

#### 2.2.2. Anti-Inflammatory Activity

TRPA1 agonist allyl isothiocyanate may directly activate the channels via covalent modification of N-terminal cysteine residues leading to the substance P secretion with a further release of inflammatory mediators [4]. Moreover, released mediators such as chemokines, bradikinin, arachidonic acid metabolites, and nitric oxide were also found to activate TRPA1 channels either directly or indirectly by means of intracellular secondary messengers [13]. Taking these facts into account, the anti-inflammatory activity of glycine esters **1**–**6** was studied on the model of AITC-induced edema. Given that non-steroidal anti-inflammatory drug ibuprofen reduces edema evoked by AITC, it has been chosen as a reference drug [19].

**Figure 1:**
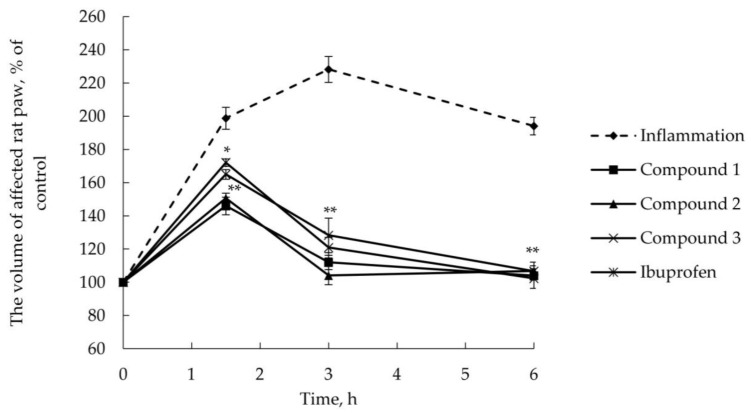
AITC-induced edema. Time dependence of the volume of affected rat paw after topical delivery (2% w/w ointment) of esters **1**–**3** and ibuprofen. * *p* < 0.05 compared with vehicle-treated group, ** *p* < 0.01 compared with vehicle-treated group. One-way analysis of variance (ANOVA) followed by Tukey’s post hoc comparison.

**Figure 2:**
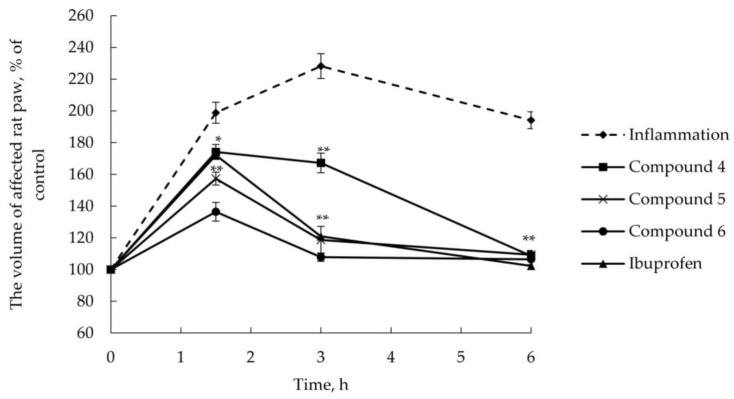
AITC-induced edema. Time dependence of the volume of affected rat paw after topical delivery (2% w/w ointment) of esters **4**–**6** and ibuprofen. * *p* < 0.05 compared with vehicle-treated group, ** *p* < 0.01 compared with vehicle-treated group. One-way analysis of variance (ANOVA) followed by Tukey’s post hoc comparison.

As illustrated in Figure 1 and Figure 2, AITC produced time-dependent increase in the volume of affected rat paw used as a measure of edema (in percentage) with a maximum at 3 h reaching the basal level (105% ± 3.9%, data not shown) at 24 h after injection.

As seen, inflammation maximum was shifted 1.5 h after AITC injection, when ibuprofen or esters **1**–**6** had been delivered transdermally (2% w/w ointment); in this case, AITC-induced edema was finished within 6 h.

Terpenoid esters **1**–**6** were shown to reduce the volume of affected rat paw after their topical application in comparison with vehicle-treated animals. Anti-inflammatory profile of synthesized compounds, excluding ester **4**, is similar and comparable to that of ibuprofen. A slightly different trend was observed for glycine ester of guaiacol (Compound **4**), which is inferior in its anti-inflammatory effect to the reference drug ibuprofen but also suppresses the inflammation induced by AITC. We may assume that this effect is due to the difference in guaiacol structure compared with other monocyclic terpenoids—the absence of additional hydrocarbon substituent in the phenolic ring. When transdermally delivered, the highest anti-inflammatory action was revealed for Compound **6**, which inhibited edema development to 136% at 1.5 h after AITC administration, while the treatment with ibuprofen ointment reduced the volume of affected paw to 172%.

Thus, the competitive inhibition between terpenoid esters and AITC for binding sites of the TRPA1 ion channel can be suggested.

## 3. Experimental Section

### 3.1. General

The following chemicals and drugs were used as obtained from their commercial suppliers: l-menthol, DMAP, glycine (Acros Organics, Geel, Belgium; Darmstadt, Germany), carvacrol, guaiacol, borneol, eugenol, DCC, di-*tert*-butyl dicarbonate (TCI, Philadelphia, PA, USA), thymol (Sigma-Aldrich, Schnelldorf, Germany). Boc-protected glycine was not obtained commercially and has been synthesized according to the literature procedure [20]. Structures of the obtained compound were established by ^1^H-NMR spectroscopy on a AVANCE DRX 500 (500 MHz) instrument (Bruker, Davis, CA, USA) using DMSO-*d*_6_ as solvent and TMS as an internal standard. FAB mass spectrum was obtained on a VG 70-70EQ mass spectrometer (VG Analytical Ltd., Manchester, UK) equipped with an Xe ion gun (8 kV); the sample was mixed with *m*-nitrobenzyl-alcohol matrix. High-resolution mass spectrometry (HRMS) was performed on a 6530 Accurate Mass quadrupole time of flight (Q-TOF) spectrometer (Agilent, Santa Clara, CA, USA) using ESI (electrospray ionization) coupled to an Agilent 1260 Infinity HPLC system. IR spectra were measured with a Frontier FT-IR spectrometer (Perkin-Elmer, Hopkinton, MA, USA) using KBr pellets. The purity and identity of the compound were monitored by TLC on Merck-made (TLC Silica gel 60 F_254_) plates (Darmstadt, Germany).

### 3.2. General Procedure for the Synthesis of GABA Esters ***1**–**6***

To a stirred solution of terpene (3.2 mmol) in CH_2_Cl_2_ (20 mL) at room temperature Boc-protected glycine (0.662 g, 3.26 mmol) and 4-dimethylaminopyridine (DMAP) (0.097 g, 0.794 mmol) were added. The reaction mixture was cooled to 0 °C, stirred for 10 min, and *N*,*N*′-dicyclohexylcarbodiimide (DCC) was added dropwise (0.727 g, 3.53 mmol). Stirring was continued for 30 min, the flask was then gradually warmed to room temperature, and the stirring continued for additional 10 h. Reaction completion was monitored by TLC. The reaction mixture was filtered, and the filtrate was diluted to 100 mL and washed with 1 M aqueous HCl, 10% aqueous NaHCO_3_, and water. Deprotection of the N-Boc group was carried out using HCl/CH_3_COOH according to the literature procedure [12]. The crude products were purified by recrystallization from methanol.

(*1R,2S,5R)-2-Isopropyl-5-methylcuclohexyl aminoacetate hydrochloride* (**1**): White solid (84%). IR ν_max_ (cm*^−^*^1^): 3460, 2953–2869, 1757, 1512, 1406, 1255. ^1^H-NMR (DMSO-*d*_6_, δ, ppm): 4.87–4.92 (td, 1H), 3.48 (s, 2H), 1.59 (m, 1H), 1,56–160 (m, 3H), 1.43–1.49 (m, 3H), 1.40 (m, 1H), 1.23–1.28 (m, 1H), 0.93 (d, *J* = 6.18 Hz, 3H), 0.86 (d, *J* = 6.71 Hz, 6H). MS (FAB) *m*/*z*: 214 [M + H]^+^. HRMS (ESI-TOF) calculated for C_12_H_24_NO_2_ [M + H]^+^ 214.3166, found 214.1446.

*2-Isopropyl-5-methylphenyl aminoacetate hydrochloride* (**2**): White solid (82%). IR ν_max_ (cm*^−^*^1^): 3326, 2933–2855, 1766, 1510, 1205, 1088, 815. ^1^H-NMR (DMSO-*d*_6_, δ, ppm): 7.25 (d, *J* = 7.03 Hz, 1H, Ar-H), 7.05 (d, *J* = 7.28 Hz, 1H, Ar-H), 6.92 (s, 1H, Ar-H), 3.63 (s, 2H, CH_2_NH_2_), 2.99–3.05 (m, 1H, CH), 2.28 (s, 3H, CH_3_), 1.13 (d, *J* = 6.98 Hz, 6H, CH_3_). MS (FAB) *m*/*z*: 208 [M + H]^+^. HRMS (ESI-TOF) calculated for C_12_H_18_NO_2_ [M + H]^+^ 208.2689, found 208.0986.

*5-Isopropyl-2-methylphenyl aminoacetate hydrochloride* (**3**): White solid (80%). IR ν_max_ (cm*^−^*^1^): 3439, 2957–2875, 1768, 1508, 1247, 1215, 908. ^1^H-NMR (DMSO-*d*_6_, δ, ppm): 7.10 (d, 2H, Ar-H), 6.98 (s, 1H, Ar-H), 3.32 (s, 2H, CH_2_NH_2_), 2.83–2.88 (m, 1H, CH), 2.11 (s, 3H, CH_3_), 1.17 (d, *J* = 6.72 Hz, 6H, CH_3_). MS (FAB) *m*/*z*: 208 [M + H]^+^. HRMS (ESI-TOF) calculated for C_12_H_18_NO_2_ [M + H]^+^ 208.2689, found 208.0986.

*2-Methoxyphenyl aminoacetate hydrochloride* (**4**): Brownish solid (74%). IR ν_max_ (cm*^−^*^1^): 3434, 3146–2935, 1734, 1500, 1413, 1178, 956. ^1^H-NMR (DMSO-*d*_6_, δ, ppm): 7.31 (t, 1H, Ar-H), 7.17 (t, 1H, Ar-H), 7.06 (d, *J* = 10.20 Hz, 1H, Ar-H), 6.70 (d, *J* = 8.60 Hz, 1H, Ar-H), 3.78 (s, 3H, CH_3_), 3.45 (s, 2H, CH_2_NH_2_). MS (FAB) *m*/*z*: 182 [M + H]^+^. HRMS (ESI-TOF) calculated for C_9_H_12_NO_3_ [M + H]^+^ 182.1886, found 182.0098.

*1,7,7-Trimethyl-bicyclo[2.2.1]heptan-2-yl aminoacetate hydrochloride* (**5**): White solid (80%). IR ν_max_ (cm*^−^*^1^): 3330, 2934–2854,1748, 1501, 1246, 1114, 1054. ^1^H-NMR (DMSO-*d*_6_, δ, ppm): 5.32–5.35 (dd, *J_1_* = 4.30 Hz, *J_2_* = 4.30 Hz, 1H, CH), 3.65 (s, 2H, CH_2_NH_2_), 2.24-2.31 (m, 1H, CH), 2.04-2.10 (m, 1H, CH), 1.88-192 (m, 1H, CH), 1.67–1.71 (m, 2H, CH), 1.38–1.44 (m, 2H, CH), 1.03 (s, 3H, CH_3_), 0.89 (s, 6H, CH_3_). MS (FAB) *m*/*z*: 212 [M + H]^+^. HRMS (ESI-TOF) calculated for C_12_H_22_NO_2_ [M + H]^+^ 212.3007, found 212.1006. 

*4-Allyl-2-methoxyphenol aminoacetate hydrochloride* (**6**): Yellowish solid (83%). IR ν_max_ (cm*^−^*^1^): 3426, 2929–2851, 1783, 1511, 1206, 1026, 902. ^1^H-NMR (DMSO-*d*_6_, δ, ppm): 7.06 (d, *J* = 6.28 Hz, 1H, Ar-H), 6.70 (s, 1H, Ar-H), 6.00 (d, *J* = 6.28 Hz, 1H, Ar-H), 5.88–5.97 (m, 1H, –CH=), 4.98 (d, *J* = 4.30 Hz, 2H, =CH_2_), 3.76 (s, 3H, CH_3_), 3.22 (d, *J* = 6.18 Hz, 2H, –CH_2_–), 3.65 (s, 2H, CH_2_NH_2_). MS (FAB) *m*/*z*: 222 [M + H]^+^. HRMS (ESI-TOF) calculated for C_12_H_16_NO_3_ [M + H]^+^ 222.2524, found 222.0986.

### 3.3. Animals

Analgesic activity of synthesized compounds was studied using outbreed male white mice (18–22 g) as experimental animals. Investigation of the anti-inflammatory effect was carried out in male Wistar rats (150–180 g). All animals were kept under a 12 h light regime and in a standard animal facility with free access to water and food, in compliance with the European Convention for the Protection of Vertebrate Animals Used for Experimental and Other Specific Purposes (Strasbourg, 1986) and the principles of the National Ukrainian Bioethics Congress (Kyiv, 2003). All animals were purchased from Odessa National Medical University, Ukraine. The Animal Ethics Committee (agreement No. 29/2016) of Odessa National University (Ukraine) approved the study.

### 3.4. Drug Administration

Topical application of esters **1**–**6** and initial monoterpenes **7**–**12** were used in order to evaluate their analgesic and anti-inflammatory effect. The ointments (2% *w*/*w*) were prepared using a mixture of polyethylene glycol (PEG 1500), polyethylene oxide (PEO 400), and 1,2-propylene glycol in the ratio of 4:2:3 as a base.

### 3.5. Antinociception Testing

Analgesic activity was measured by the “hot plate” test as an acute pain model according to a procedure found in [17]. The mice were placed on a hot plate maintained at 55 °C one at a time. In this experiment, latency to respond to the heat stimulus was determined by the amount of time (in seconds) it takes for a given mouse to lick one of its paws. Cut-off time was fixed at 60 s to minimize the tissue damage that occurs during prolonged contact with heated surface.

The antinociceptive activity was also evaluated by models of chemical stimuli with the use of the TRPA1 agonists formalin, allyl isothiocyanate (AITC), and TRPV1 agonist capsaicin. The method used was similar to that described in [21]. Following the adaptation to the experimental conditions, 20 μL 2% formalin solution in 0.9% NaCl/20 μL of 0.5% (*w*/*w*) AITC/20 μL of capsaicin (6 μg/paw) solution were injected subcutaneously under the skin of the dorsal surface of the right hindpaw. The ointments were applied to the plantar surface of the right paw by gently rubbing 5 min before antinociceptive activity evaluation. Control animals received only a corresponding amount of the ointment base. AITC and capsaicin doses were selected based on our preliminary trials and from literature citations. Animals were observed individually for 5/10/5 min, respectively, after formalin/AITC/capsaicin administration. The amount of time that mice spent licking their respective injected paws (reaction time in seconds) was recorded and considered as an index of pain.

### 3.6. AITC-Induced Acute Inflammatory Model

Anti-inflammatory activity of Compounds **1**–**6** and reference drug ibuprofen was determined using an AITC-induced rat paw edema assay. Edema was induced by subplantar injection of 30 μL of AITC solution (100 μg/paw) in propylene glycol 30 min prior to ointment application. Edema was measured before (control data) and at 1.5, 3, 6, and 24 h after AITC injection by using a plethysmometer. The ointments were additionally applied by gently rubbing on the affected paw at each time point. Values of paw volume at each time point are expressed as a percentage relative to control (non-inflamed paw).

### 3.7. Statistical Analysis

All results are expressed as mean ± standard error mean (SEM). One-way analysis of variance (ANOVA) was used to determine the statistical significance of the results followed by Tukey’s post hoc comparison. *p* < 0.05 was considered significant.

## 4. Conclusions

We have synthesized esters based on mono- and bicyclic terpenoids (L-menthol, thymol, carvacrol, guaiacol, borneol, and eugenol) with a neurotransmitter acid—glycine. Analgesic activity of obtained compounds was investigated by pharmacological models of thermal and chemical stimuli in mice after their transdermal delivery. Herein, we propose competitive binding between terpenoid esters and TRPA1/TRPV1 agonists as an explanation of the significant analgesic effect of terpene esters. For clarification of high anti-inflammatory activity of terpene derivatives, we suggest competitive inhibition between terpenoid esters and AITC for binding sites of the TRPA1 ion channel.

## Figures and Tables

**Table 1:** Analgesic activity of Compounds **1**−**6** in comparison with initial terpenoids **7**−**12** tested by the hot plate method in mice (2% w/w ointment).

Compound	Reaction Time (s)	Compound	Reaction Time (s)
Control	10 ± 0.6	**6**	32 ± 3.8 **
Benzocaine	18 ± 0.9 *	**7**	24 ± 3.9 *
**1**	29 ± 6.5 *	**8**	15 ± 0.3 *
**2**	47 ± 1.3 **	**9**	19 ± 1.9 *
**3**	21 ± 2.5 *	**10**	20 ± 0.5 *
**4**	23 ± 2.8 *	**11**	27 ± 2.8 *
**5**	49 ± 0.9 **	**12**	21 ± 3.1 *

All values are expressed as mean ± SEM; *n* = 6; * *p* < 0.05 compared with control group, ** *p* < 0.01 compared with benzocaine. One-way analysis of variance (ANOVA) followed by Tukey’s post hoc comparison.

**Table 2:** Analgesic activity of Compounds **1**−**6** in comparison with initial terpenoids **7**−**12** on Phase I of the formalin test in mice (2% w/w ointment).

Compound	Licking Time (s)	Compound	Licking Time (s)
Control	103 ± 8.5	**6**	30 ± 5.6
Benzocaine	36 ± 2.4	**7**	24 ± 5.6
**1**	19 ± 3.7	**8**	50 ± 5.2
**2**	47 ± 1.3	**9**	38 ± 3.8
**3**	29 ± 6.9	**10**	54 ± 6.8
**4**	35 ± 1.9	**11**	26 ± 4.7
**5**	19 ± 1.5	**12**	40 ± 3.3

All values are expressed as mean ± SEM; *n* = 6; for all groups *p* < 0.01 compared with control. One-way analysis of variance (ANOVA) followed by Tukey’s *post hoc* comparison.

**Table 3:** Analgesic activity of Compounds **1**−**6** in comparison with initial terpenoids **7**−**12** on AITC-induced acute pain in mice (2% w/w ointment).

Compound	Licking Time (s)	Compound	Licking Time (s)
Control	71 ± 1.8	**6**	25 ± 1.2 *
Benzocaine	48 ± 2.0 *	**7**	7 ± 1.2 **
**1**	16 ± 4.6 **	**8**	25 ± 3.8 *
**2**	13 ± 0.9 **	**9**	35 ± 2.2 *
**3**	15 ± 0.9 **	**10**	21 ± 2.8 *
**4**	30 ± 8.3 *	**11**	8 ± 3.5 **
**5**	20 ± 1.2 **	**12**	30 ± 2.2 *

All values are expressed as mean ± SEM; *n* = 6; * *p* < 0.05 compared with control group, ** *p* < 0.01 compared with benzocaine. One-way analysis of variance (ANOVA) followed by Tukey’s post hoc comparison.

**Table 4:** Analgesic activity of Compounds **1**−**6** in comparison with initial terpenoids **7**−**12** on capsaicin-induced acute pain in mice (2% w/w ointment).

Compound	Licking Time (s)	Compound	Licking Time (s)
Control	46 ± 1.8	**6**	18 ± 0.9 *
Benzocaine	29 ± 6.6 *	**7**	11 ± 2.7 **
**1**	11 ± 1.5 **	**8**	35 ± 4.1
**2**	16 ± 3.2 *	**9**	23 ± 6.4 *
**3**	14 ± 0.8 *	**10**	23 ± 2.7 *
**4**	20 ± 3.6 *	**11**	12 ± 5.0 *
**5**	15 ± 1.3 *	**12**	20 ± 3.4 *

All values are expressed as mean ± SEM; *n* = 6; * *p* < 0.05 compared with control group, ** *p* < 0.01 compared with benzocaine. One-way analysis of variance (ANOVA) followed by Tukey’s post hoc comparison.
